# Acquisition and clearance of multidrug resistant *Acinetobacter baumannii* on healthy young adults concurrently burned in a dust explosion in Taiwan: the implication for antimicrobial stewardship

**DOI:** 10.1186/s12879-017-2682-4

**Published:** 2017-08-30

**Authors:** Po-Yen Huang, Shian-Sen Shie, Jung-Jr Ye, Shih-Pin Lin, Tsui-Ping Liu, Ting-Shu Wu, Tsu-Lan Wu, Shiow-Shuh Chuang, Ming-Huei Cheng, Yu-Chia Hsieh, Ching-Tai Huang

**Affiliations:** 1 Division of Infectious Diseases, Department of Medicine, Chang Gung Memorial Hospital and Chang Gung University, 5 Fu-Shin St., Kweishan, 333 Taoyuan, Taiwan; 2 Infection Control Committee, Chang Gung Memorial Hospital and Chang Gung University, Taoyuan, Taiwan; 3 Division of Biostatistics, Graduate Institute of Epidemiology and Preventive Medicine, National Taiwan University, Taipei, Taiwan; 4 Department of Anesthesiology, Taipei Veterans General Hospital and National Yang-Ming University School of Medicine, Taipei, Taiwan; 5 Department of Laboratory Medicine, Chang Gung Memorial Hospital and Chang Gung University, Taoyuan, Taiwan; 6 Department of Plastic and Reconstructive Surgery, Chang Gung Memorial Hospital and Chang Gung University, Taoyuan, Taiwan; 7 Division of Pediatric Infectious Diseases, Department of Pediatrics, Chang Gung Children’s Hospital, Chang Gung University College of Medicine, 5 Fu-Shin St., Kweishan, 333 Taoyuan, Taiwan

**Keywords:** *Acinetobacter Baumannii*, Burn patients, Antibiotic resistance, Antibiotic stewardship, Carbapenem

## Abstract

**Background:**

Information is limited about the effect of restricted carbapenem use on clearance of multi-drug resistant *Acinetobacter baumannii* (MDRAB). We sought to determine the time effect of antibiotic exposure on multi-drug resistant *Acinetobacter baumannii* (MDRAB) acquisition and clearance.

**Methods:**

We conducted a retrospective observational study at the intensive care units of a tertiary medical center. Forty-two of a cohort of previously healthy young adults who were concurrently burned by a dust explosion was included. Cases consisted of those from whom MDRAB was isolated during hospitalization. Controls consisted of patients from whom MDRAB was not isolated in the same period. Use of antimicrobial agents was compared based on days of therapy per 1,000 patient-days (DOT/1,000PD). A 2-state Markov multi-state model was used to estimate the risk of acquisition and clearance of MDRAB.

**Results:**

MDRAB was discovered in 9/42 (21.4%) individuals. The cases had significantly higher use of carbapenem (652 DOT/1,000PD vs. 385 DOT/1,000PD, *P* < 0.001) before MDRAB isolation. For the cases, clearance of MDRAB was associated with lower use of carbapenem (469 DOT/1,000PD vs. 708 DOT/1,000PD, *P* = 0.003) and higher use of non-carbapenem beta-lactam (612 DOT/1,000PD vs. 246 DOT/1,000PD, *P* <0.001). In multi-state model, each additional DOT of carbapenem increased the hazard of acquiring MDRAB (hazard ratio (HR), 1.08; 95% confidence interval (CI) 1.01–1.16) and each additional DOT of non-carbapenem beta-lactam increased the protection of clearing MDRAB (HR, 1.25; 95% CI 1.07–1.46).

**Conclusions:**

Both acquisition and clearance of MDRAB were related to antibiotic exposure in a homogeneous population. Our findings suggest that early discontinuation of carbapenem could be an effective measure in antibiotic stewardship for the control of MDRAB spreading.

## Background

*Acinetobacter baumannii* is an important pathogen, causing healthcare-associated infections such as pneumonia, urinary tract infection, soft tissue infection, and bloodstream infection [1, 2]. The extraordinary metabolic versatility of *A. baumannii* contributes to this organism’s survival in the environment, including persistence for extended periods of time on dry surfaces [3]. Given this organism’s propensity to horizontally acquire resistance to multiple classes of antimicrobial agents, *A. baumannii* ranks among the most important nosocomial pathogens [4]. The increasing spread of international clones of multidrug-resistant *Acinetobacter baumannii* (MDRAB) with decreased susceptibility to carbapenem poses a great threat to the health care system; alternative therapeutic options are limited and outcome is poor in patients infected by beta-lactam-resistant *A. baumannii* [5, 6]. An integrated, multidisciplinary approach is advocated to control the growing threat of MDRAB-related infection or colonization. Proposed responses have included changes in hand hygiene, surveillance, cohort policy, environmental disinfection and cleaning, contact isolation, decolonization, the use of chlorhexidine baths, and antibiotic stewardship programs (ASPs), all with the intent of eliminating this bacterium’s reservoir, transmission, and source [7–10].

Studies have shown that antimicrobial use is associated with the emergence of drug-resistant pathogens; the implementation of an ASP may be effective in reducing resistance rates [11–17]. While there is increasing evidence that MDRAB acquisition may be related to prior carbapenem exposure [18–20], there is typically a time lag of one to several months between antimicrobial prescription and the emergence of bacterial resistance [16, 21]. The most affected patients are those of advanced age, immunosuppressed status, and/or comorbidities that create a need for broader antimicrobial coverage in clinical situations. However, the direct effect of restricted use of carbapenem at the individual patient-level remains poorly understood.

On June 27, 2015, a dust explosion occurred in the late evening at the Formosa Fun Coast water park in northern Taiwan, creating over 400 burn victims and representing a major challenge to the health care system [22]. Among the victims were forty-two previously healthy young adults who presented with severe burns and were admitted as a cohort to two intensive care units (ICUs) at Chang Gung Memorial Hospital. The dust explosion was caused by a flammable starch-based colored powder. The powder created an extremely dense dust cloud, which immediately caused partygoers to be engulfed by flames when the powder ignited accidentally [23]. This event therefore provided with an opportunity to study the effect of exposure to various antibiotics on the acquisition and clearance of MDRAB in a homogeneous group of patients.

## Methods

### Setting, study design, and the patients

This case-control study was conducted at the Chang Gung Memorial Hospital-Linkou Branch, a 3700-bed tertiary-care medical center with 308 ICU beds for critical care in northern Taiwan. The facility has two ICUs (48 beds) which were devoted exclusively to care of critically ill burn patients. The present investigation included 42 previously healthy young adults who had been admitted to the ICUs between June 27, 2015 and July 1, 2015 due to the dust burn occurred on June 27, 2015 in Taiwan. This analysis included all inpatient data until discharge or death. The study was approved by the Institutional Review Board of Chang Gung Memorial Hospital (104-9356B) and informed consent was waived due to the study’s retrospective nature.

The key managements for the flame burn were accomplished as previously described [23]. In addition, microbiological surveillance was performed during hospitalization. All these burned young adults underwent wound cultures twice a week, tissue cultures whenever surgical debridement was performed. Blood cultures or urine cultures were done if fever and/or sepsis was suspected. Central venous catheter (CVC) would be removed and culture from a CVC tip was performed whenever CVC related infection was suspected. Standard precautions and contact precautions were applied to every patient. Aprons and gloves were used regularly. For appropriate use of antimicrobial agents, a comprehensive ASP has implemented since 2005 at the hospital, with satisfactory outcomes [13, 15]. The stewardship program involves multidisciplinary professionals including physicians, pharmacist, infection control nurses, as well as policy makers. It is a web-based healthcare information system with prospective audit and online feedback. Both the infectious disease physicians and the pharmacists will review the antimicrobial prescriptions within 48 h. Cases were defined as those from whom MDRAB was isolated from any site during hospitalization. Controls were defined as patients from whom MDRAB was not isolated during hospitalization. For the cases, clearance of MDRAB was defined as two consecutive surveillance cultures of colonized/infected sites negative for MDRAB.

### Differentiation between colonization and infection

Initially we tried to distinguish between infection and colonization status of the patients. However, after reviewing the charts, we found that we could not clearly define infection or colonization status regarding isolation of MDRAB based on objective data. The patients shared similar clinical findings, laboratory data, and isolates that were collected intraoperatively. Thus, we defined the condition of the patients as MDRAB infection/colonization.

### Data collection and measurement of antimicrobial use

Demographics, underlying diseases, ventilator use, inhalation injury, and catheter use were reviewed. For evaluation of disease severity, an Acute Physiology And Chronic Health Evaluation (APACHE) II score was determined on the first day of admission. To quantify antimicrobial use, the prescriptions of each patient was reviewed and converted into days of therapy (DOTs). We defined the DOT as previously described [24]. In short, one DOT represents the administration of a single agent on a day, without consideration of the number of doses or the strength of dosage. Difference in antimicrobial use was calculated by DOT per 1000 patient-days (DOT/1000PD), as an adjusted rate of antimicrobial exposure. We also compared the antimicrobial use based on defined daily dose (DDD) and DDD per 1000 patient-days (DDD/1000PD) between the cases and the controls.

### Time at risk and comparisons of antimicrobial use

For the cases with MDRAB, the time-at-risk was calculated as the number of days from the date of admission to the date of MDRAB detection. For the controls without MDRAB isolation during hospitalization, the time-at-risk was calculated as for the cases starting on the date of admission. For the evaluation of clearance of MDRAB, the time-at-risk was the number of days between MDRAB isolation and MDRAB clearance. The adjusted rate of antimicrobial use (DOT/1000PD) was then calculated and compared between 1) the cases and the controls, and 2) before and after MDRAB isolation.

### Identification of multidrug-resistant *Acinetobacter baumannii*

Identification of *A. baumannii* was performed by Matrix-Assisted Laser Desorption-Time Of Flight (MALDI-TOF) mass spectrometry (MS). Susceptibility to all tested antibiotics except tigecycline was determined according to the Clinical and Laboratory Standards Institute (CLSI) interpretive criteria for disk diffusion method. Susceptibility to tigecycline was determined using the disk diffusion method with Mueller-Hinton agar (BD Microbiology Systems, Cockeysville, MD), with breakpoints at ≧ 16 mm and ≦ 12 mm. MDRAB was defined as *A. baumannii* with full or intermediate resistance to amikacin, gentamicin, cefepime, ceftazidime, piperacillin, piperacillin-tazobactam, aztreonam, ciprofloxacin, and carbapenems [18].

### Statistical analysis

We used a multistate model analyzing the process of acquisition and clearance of the MDRAB [25, 26]. The multistate model described the two states (MDRAB negative and MDRAB positive) of the patients and also allowed estimation of the transition rate (acquisition and clearance) between the two states. When MDRAB was isolated from a given patient’s clinical specimens, the patient was considered to have transitioned from the negative state to the positive state (MDRAB acquisition). The multistate model used a transition intensity matrix to model the transition rate between the two states. Another detail of the multistate model included the assumption that each transition between states followed a “no memory” property that could be described as an exponential distribution. When analyzing the effect of covariates, the Cox proportional hazard regression model was used. The significance of a covariate was determined using the Wald test statistic, which was calculated as the difference in the transition probability between groups divided by the standard error (square root of the sum of the 2 variances). The effects of covariates on the transition rates were quantified as the hazard ratio (HR), with a ratio greater than 1 indicating a positive effect on the transition rate. Other continuous variables were compared using Student’s t test or the Mann-Whitney U test. Binomial variables were compared using chi-squared or Fisher’s exact test. A 2-tailed *p* value <0.05 was considered statistically significant in all tests. All statistical analyses were performed with SAS software (V9.4; SAS Institute, Inc., Cary, NC, USA).

## Results

MDRAB occurred in nine of forty-two patients during the study period (21.4%). In most (8 of 9) of these instances, MDRAB was detected from wounds. In only one instance was MDRAB isolated from the tip of a removed central venous catheter (CVC). Compared to the control group, the case group had similar demographics, total body surface area (TBSA) affected by burn, and APACHE II score on admission. No comorbid illness was reported in either group of patients. The median day to MDRAB occurrence was 16 days. Acquisition of MDRAB was associated with higher use of carbapenem (652 DOT/1000PD vs. 385 DOT/1000PD, *P* < 0.001), lower use of non-carbapenem beta-lactam (298 DOT/1000PD vs. 534 DOT/1000PD, *P* < 0.001). There was no difference of overall antibiotic use between the case group and the control group (1929 DOT/1000PD vs. 1890 DOT/1000PD, *P* = 0.759). Measurement of DOT was comparable with that of DDD (correlation coefficient = 0.94) (Table 1).

**Table 1:** Demographics and antimicrobial use of the healthy young adults admitted due to a dust explosion with or without MDRAB occurrence

	MDRAB (*n* = 9)	No MDRAB (*n* = 33)	*P* value
Age, median year (IQR)	19 (18–21)	21 (19–24)	0.275
Male, no. (%)	2 (22)	11 (49)	0.258
TBSA, %	50 (40–60)	45 (30–58)	0.460
APACHE II score (IQR)	6 (4–7)	6 (4–7)	0.409
Inhalation injury	2 (22)	14 (44)	0.442
Mechanical ventilation on admission, no. (%)	5 (56)	22 (67)	0.698
Mechanical ventilation at week 1, no. (%)	6 (67)	25 (76)	0.676
Mechanical ventilation at week 2, no. (%)	5 (56)	21 (64)	0.711
LOS before MDRAB occurrence, day (IQR)	16 (11–18)	NA	NA
Hospital day, median day (IQR)	93 (48–112)	53 (34–79)	0.018
Bacteremia, no. (%)	2 (22)	10 (24)	1.000
In-hospital mortality, no. (%)	0 (0)	2 (6%)	1.000
Antimicrobial exposure^a^			
Time-at-risk, patient-days^b^	141	517	
All antibiotics, DOT (DOT/1000 patient-days)	272 (1929)	977 (1890)	0.759
Carbapenem	92 (652)	199 (385)	< 0.001
Non-carbapenem Beta-lactam	42 (298)	276 (534)	< 0.001
Glycopeptide	116 (823)	384 (743)	0.335
Miscellaneous	22 (156)	118 (228)	0.093
Antifungal agent, DOT (DOT/1000 patient-days)	51 (361)	153 (296)	0.218
All antibiotics, DDD (DDD/1000 patient-days)	299 (2120)	1099 (2126)	0.975
Carbapenem	91 (642)	187 (362)	< 0.001
Non-carbapenem Beta-lactam	61 (431)	360 (695)	< 0.001
Glycopeptide	126 (893)	427 (826)	0.435
Miscellaneous	22 (153)	125 (243)	0.050
Antifungal agent, DDD (DDD/1000 patient-days)	81 (574)	276 (533)	0.557

Data are expressed as median and interquartile range or number (%) of patients unless otherwise indicated

^a^Antimicrobial exposure is expressed as days of therapy (DOTs) and accumulated DOTs per 1000 patient-days

^b^Time-at-risk for the controls was calculated as for the cases starting on the date of admission in a 1:3 or 1:4 case-control match. The time-at-risk of those controls were matched with that of the cases, if possible

*APACHE* Acute Physiology and Chronic Health Evaluation, *DDD* defined daily dose, *DOT* days of therapy, *IQR* interquartile range, *LOS* length of stay, *MDRAB* multidrug-resistant *Acinetobacter baumannii*, *NA* not applicable, *TBSA* total body surface area

Antimicrobial use was compared before and after the acquisition of MDRAB in the case group (Table 2). The analysis excluded one patient because the MDRAB was isolated from a CVC tip and clearance could not be defined. Antimicrobial exposure differed significantly in carbapenem, non-carbapenem beta-lactam antibiotics, and miscellaneous antibiotics. There was no difference in use of glycopeptide. Clearance of MDRAB was associated with fewer carbapenem use after adjusted with time-at-risk (469 DOT/1000PD vs. 708 DOT/1000PD, *P* = 0.003). Characteristics, outcomes, as well as detailed antimicrobial use before and after the isolation of MDRAB in the cases (*N* = 9) were compared. Four of whom have stopped carbapenem and received ceftazidime after the MDRAB occurrence. Three of whom have continued carbapenem use. Two of whom have continued non-carbapenem beta-lactam use (Table 3).

**Table 2:** Comparison of antimicrobial use among the eight^a^ MDRAB cases before and after the occurrence of MDRAB

	Before	After	*P* value
Time-at-risk, patient-days^b^	130	98	
All antibiotics, DOT (DOT/1000 patient-days)	1923 (1685–2161)	2286 (1986–2585)	0.113
Carbapenem	708 (563–852)	469 (334–605)	0.003
Non-carbapenem Beta-lactam	246 (161–331)	612 (457–767)	< 0.001
Glycopeptide	846 (688–1004)	939 (747–1131)	0.424
Miscellaneous	123 (63–183)	265 (163–367)	0.016
Antifungal agent	392 (285–500)	765 (592–939)	< 0.001

^a^One of the nine patients was excluded from the analysis. For this patient, only one MDRAB isolate was discovered from a removed central venous catheter (CVC) and there was no further MDRAB isolation from CVC available during study period. Thus, clearance of MDRAB was not defined and the analysis did not include the patient

^b^Time-at-risk before MDRAB occurrence was the time from admission to MDRAB isolation. Time-at-risk after MDRAB occurrence was the time from MDRAB isolation to MDRAB clearance

Data are expressed as days of therapy (DOTs) and accumulated DOTs per 1000 patient-days

*DOT* days of therapy, *MDRAB* multidrug-resistant *Acinetobacter baumannii*

**Table 3:** Characteristics of the nine patients with multi-drug resistant *Acinetobacter baumannii* isolation

No.	Source	Concurrent bacteremia	APACHE II score	TBSA (%)	LOS before MDRAB occurrence	Days from MDRAB occurrence to clearance	Antimicrobial use before MDRAB isolation	Antimicrobial use after MDRAB isolation
1	Wound	no	6	40	16	10	Teicoplanin, imipenem	Teicoplanin, ceftazidime
2	Wound	no	4	60	15	14	Teicoplanin, imipenem	Teicoplanin, ceftazidime
3	Wound	no	7	40	22	9	Teicoplanin, imipenem	Teicoplanin, meropenem
4	CVC	no	4	35	11	NA	Teicoplanin, ceftazidime	Teicoplanin, ceftazidime
5	Wound	*Klebsiella pneumoniae* *Staphylococcus haemolyticus*	9	55	11	21	Teicoplanin, imipenem	Daptomycin, imipenem
6	Wound	no	6	60	26	10	Teicoplanin, ceftazidime	Cefepime
7	Wound	*Acinetobacter pittii* *Staphylococcus haemolyticus*	13	80	16	14	Teicoplanin, imipenem	Teicoplanin + impenem + colistin, teicoplanin + cefaperazone/sulbactam
8	Wound	no	6	50	18	10	Teicoplanin, imipenem	Teicoplanin + ceftazidime
9	Wound	no	4	40	6	10	Teicoplanin, ceftazidime	Teicoplanin + cefaperazone/sulbactam

MDRAB was cleared before discharge for all the patients except one person (No. 4) who had a removed central venous catheter (CVC) with MDRAB detection. All the cases survived and were discharged from the hospital uneventfully

*CVC* central venous catheter, *LOS* length of stay, *MDRAB* multi-drug resistant *Acinetobacter baumannii*, *NA* not applicable, *TBSA* total body surface area

**Fig. 1:**
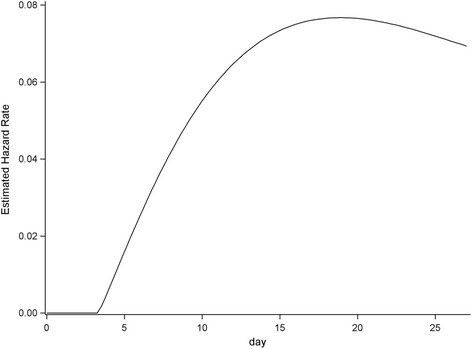
The overall hazard rate of acquiring MDRAB in the first 28 days after admission among the study population

In the null model without considering any clinical correlates, the rate of transition from MDRAB negative to MDRAB positive (MDRAB acquisition) was 0.005, while the rate of transition from MDRAB positive to MDRAB negative (MDRAB clearance) was 0.01. The risk of acquiring MDRAB sharply increased 3 days after admission (Fig. 1). Antibiotic exposure had a significant effect on the transition between MDRAB negative and MDRAB positive (Table 4). In univariate analysis, carbapenem significantly affected the rate of MDRAB acquisition. Every additional carbapenem DOT increased the hazard of acquiring MDRAB by 9% (HR, 1.09; 95% CI 1.02–1.16; *P* = 0.007) (Table 4). Glycopeptide, non-carbapenem beta-lactam, and antifungal agents showed no significant effect on MDRAB acquisition. Regarding clearance of MDRAB, every additional non-carbapenem beta-lactam DOT increased the protection of clearing MDRAB by 19% (HR, 1.19; 95% CI 1.05–1.34; *P* = 0.024) (Table 4). Carbapenem, glycopeptide, and antifungal agents showed no significant effect on MDRAB clearance. In multivariate analysis, carbapenem retained a positive effect on MDRAB acquisition (HR, 1.08; 95% CI 1.01–1.16; *P* = 0.020) as did non-carbapenem beta-lactam on MDRAB clearance (HR, 1.25; 95% CI 1.07–1.46; *P* = 0.004) (Table 4).

**Table 4:** Hazard ratios for the acquisition and clearance of MDRAB

Variable	Univariate analysis				Multivariate analysis			
	acquisition hazard ratio, (95% CI)	*P value*	clearance hazard ratio, (95% CI)	*P value*	acquisition hazard ratio, (95% CI)	*P value*	clearance hazard ratio, (95% CI)	*P value*
Baseline transition rate (/day)					0.005		0.01	
Age	0.90 (0.74–1.09)	0.271	1.10 (0.91–1.33)	0.318				
Sex	0.41 (0.08–1.98)	0.268	2.38 (0.49–11.46)	0.281				
Cumulative antibiotic exposure (days of therapy)								
Carbapenem	*1.09 (1.02-1.16)* ^b^	0.007	1.0 (0.94–1.06)	0.908	*1.08 (1.01-1.16)* ^b^	0.020	1.07 (0.99–1.17)	0.083
Glycopeptide	1.04 (0.98–1.11)	0.209	1.1 (1.0–1.2)	0.140	-		-	
Non-carbapenem Beta-lactam	0.9 (0.78–1.100)	0.340	*1.19 (1.05-1.34)* ^b^	0.024	0.97 (0.81–1.15)	0.711	*1.25 (1.07-1.46)* ^b^	0.004
Antifungus	1.02 (0.94–1.1)	0.594	1.1 (0.97–1.1)	0.196	-		-	
Miscellaneous	^a^		^a^		-		-	

^a^indicated too small sample size for model estimation

^b^Hazard ratio with statistical significance

## Discussion

We conducted this study to examine the association between carbapenem and MDRAB in an extremely homogeneous group of previously healthy young burn patients. Our results show that antibiotic exposure was related to both the acquisition and clearance of MDRAB. In particularly, MDRAB clearance was associated with restricted use of carbapenem. Each additional DOT of carbapenem increased the risk of MDRAB acquisition, and each additional DOT of non-carbapenem beta-lactam increased the chance of MDRAB clearance. Our findings highlight the impact of antimicrobial use on the occurrence of drug-resistant pathogens, suggesting that the early discontinuation of carbapenem use may help to control the emergence of MDRAB.

Munoz-Price et al. [27] reported that every additional defined daily dose (DDD) of carbapenem increased the risk of carbapenem-resistant *A. baumannii* colonization (among patients initially found to be non-colonized) by 5.1%. In the group of previously healthy young burn patients, we demonstrated that carbapenem was a time-dependent variable for MDRAB acquisition, and every additional DOT increased the risk of MDRAB acquisition by 8%. Both the studies explored the effect of carbapenem exposure as a continuous variable to further understand the association between time-dependent antibiotic exposure and acquisition of MDRAB. However, our cohort is uniquely positioned to evaluate the impact of antibiotic exposure on the occurrence of MDRAB infection/colonization, given that our patients were free of underlying conditions and were admitted at the same time due to the dust explosion; the results of these experiments provided additional data to facilitate the optimization of antibiotic prescription by clinicians.

The importance of detection of carbapenem-resistant *A. baumannii* colonization lies in the high predictive value with regard to the subsequent development of carbapenem-resistant *A. baumannii* infection [28]. Notably, infection by carbapenem-resistant *A. baumannii* is associated with a mortality rate of approximately 52%, compared with a 19% mortality rate observed following infection with carbapenem-susceptible *A. baumannii* [29]. Our findings support the short-term impact of an ASP on the clearance of drug-resistant pathogens; the early discontinued use of carbapenem may help to control an MDRAB outbreak. In the long term, carbapenem use may increase following the implementation of ASP due to the ballooning effect [15]. Carbapenems, which exhibit broad-spectrum activity against bacteria, may impose collateral damage and perturb the human indigenous microbiota [30]. Halting carbapenem use may be helpful for reframing and restoring the human microbiome, which may combat MDRAB, and for precluding the selection of resistant strains from a population of susceptible bacteria. Our results also suggest that the competitive advantage of *A. baumannii* in the microbial ecology may solely reflect this organism’s resistance to antimicrobial agents under selective pressure.

There has been a report that beta-lactam was a risk factor for acquisition of MDRAB [31]. Furthermore, differing from our findings, the usage of carbapenem tended to correlate to clearance of MDRAB in the multivariable analysis with borderline significance [31]. One possible reason is that our patient group was comprised of a cohort of relatively young burn victims without comorbid illnesses. There were few potential confounding variables. Most of the case patients (7/9) were cleared from MDRAB without antimicrobial therapy directed against MDRAB (Table 3). Still, the patients received other antibiotics due to critically ill conditions and burn injury. There were two of the nine cases who received specific treatment for MDRAB (Case No. 7 and case No. 9 in Table 3). Despite limited number of cases, our findings may imply the possibility of spontaneously clearance of MDRAB in immune competent hosts. It might be partly caused by less selective pressure and the clearance could occur without pharmacological intervention.

Both DDD and DOT are standardized methods for measurement of antibiotic use [32]. We chose DOT divided by time-at-risk (i.e., accumulated person days) to compare the rate of antimicrobial use between the case and the control group. As the measure of antimicrobial use that best predicts the prevalence of antimicrobial resistance has not yet been defined [33], one advantage of DOT is that it is not affected by changes in dosing regimen [21]. Moreover, DOT could be more helpful in comparing the use of different classes of antimicrobials within a given institution and indicate timely antimicrobial usage in a particular patient [21]. Because the clinicians might adjust the antimicrobial regimens promptly when treating critically ill patients, we think that measurement of the DOTs would be a better reflection of clinical scenario and usage of antimicrobial agents.

Our study does have some limitations. Due to this study’s retrospective nature and single-center design, our results may be not applicable in other settings or hospitals. There were more than 400 injuries due to the dust explosion and only 42 of the them who were admitted to the ICUs of the hospital were included. This has implications for possible selection bias. However, our study population consisted of young adults who were previously healthy and admitted as a concurrent group following a single event (the dust explosion). The patients were admitted to the hospital at the same time between June 27, 2015 and July 1, 2015. We acknowledged that medical procedures and environmental factors played important roles in the process of MDRAB colonization. The integrated and intensive care provided by the hospital at that time maximally eliminated the confounding factors that were measurable [23]. Hence, the results are expected to be relevant and may contribute to clinical decision-making. Another potential drawback is that we did not investigate the indications of antimicrobial use in our analysis. However, a well-established integrated ASP has been implemented in the hospital for a decade; each prescription was carefully reviewed daily by both pharmacists and infection diseases specialists [10, 12]. As a result, decisions regarding antimicrobial use were attributable to responsible clinicians; appropriate use of antimicrobial agents could be expected in the study. The definition of MDRAB clearance among the patients with prior MDRAB isolation may be concerned. We defined MDRAB clearance according to the hospital’s policy, which requires surveillance cultures to be obtained within 7 days of the previous culture. By definition, eight of nine MDRAB-positive patients were clear from MDRAB. Biases were minimized, given that the ASP were applied regularly and consistently across the hospital. A further limitation of our study was that we did not perform contemporaneous environmental surveillances in either of the two ICUs. This precluded the analysis of interactions between the patients and their hospital environment. Finally, we did not discern between infection and colonization status in the study. Yet the conclusion may be valid in either infection or colonization status.

## Conclusions

In conclusion, our results suggest that antibiotic exposure is associated with both acquisition and clearance of MDRAB in this homogeneous population of previously healthy, critically ill young burn patients. Our findings reinforce the utility of an ASP in reducing the occurrence of multidrug-resistant pathogens, and highlight the appropriateness of early discontinuation of carbapenem when the use of carbapenem is inevitable.

## Abbreviations

ASP: antibiotic stewardship program
CI: Confidence interval
CVC: central venous catheter
DDD: Defined daily dose
DOT: Day of therapy
HR: Hazard ratio
ICU: Intensive care unit
MDRAB: Multidrug-resistant *Acinetobacter baumannii*
TBSA: total body surface area
